# Confluent and Reticulated Papillomatosis Associated With Obesity: Case Series of Three Patients Successfully Treated With Oral Doxycycline

**DOI:** 10.5826/dpc.1102a06

**Published:** 2021-04-12

**Authors:** Maha Lahouel, Amina Aounallah, Sana Mokni, Colandane Belajouza, Mohamed Denguezli

**Affiliations:** 1 Department of Dermatology, Farhat Hached University Hospital, Sousse, Tunisia

**Keywords:** confluent and reticulated papillomatosis, obesity, treatment, doxycycline

## Introduction

Confluent and reticulated papillomatosis (CRP) of Gougerot and Carteaud is a rare skin disorder [1,2]. Its pathogenesis remains unclear, which explains the multiplicity of therapy options with variable results [1]. Herein, we describe 3 cases of CRP, discuss its association with obesity, and report the effectiveness of doxycycline in its treatment.

## Case Presentations

### Case 1

**Figure 1:**
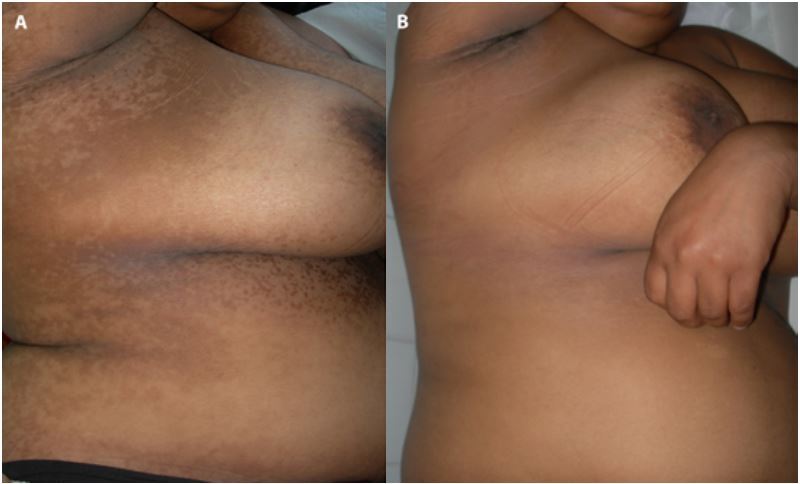
(A) Brownish, confluent papules with a slightly hyperkeratotic surface that affected mainly the trunk. (B) Complete clearance of the eruption after 2 months of treatment with doxycycline.

A 20-year-old woman presented with hypertrophic squamous lesions mainly localized on the trunk that had been evolving for the previous 2 years. At presentation, the patient had a body mass index (BMI) of 35.9 (obesity class II). Examination revealed brownish, hyperkeratotic, confluent papules with a reticulated pattern in the periphery affecting mainly the trunk (Figure 1A), the neck and the back. A biopsy demonstrated compact hyperkeratosis, acanthosis, papillomatosis, and hyperpigmentation of the basal layer. The diagnosis of CRP was made. Doxycycline was initiated (100 mg daily). Two months later, the patient was free of cutaneous lesions (Figure 1B). The patient’s skin condition was stable after 1 year of follow-up.

### Case 2

**Figure 2:**
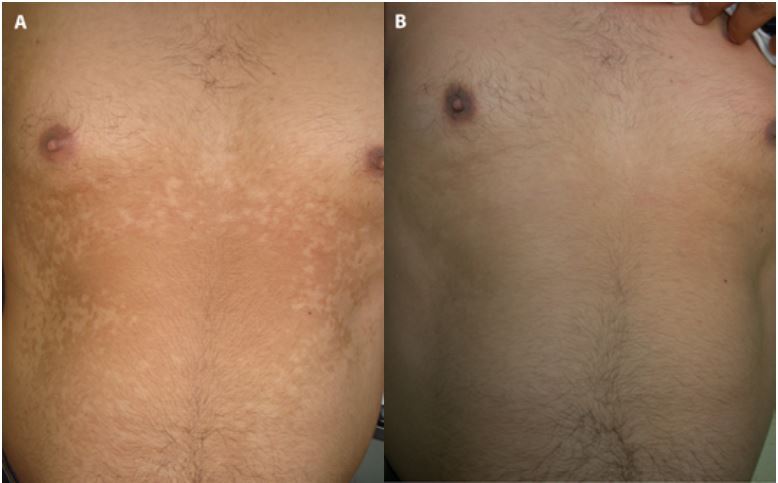
(A) Asymptomatic hyperpigmented papules and plaques with a reticulated pattern. (B) Complete resolution of lesions within 1 month of doxycycline treatment.

A 21-year-old overweight man (BMI >25) presented with a 5-month history of asymptomatic lesions on the trunk. On dermatologic examination, there was a reticular network of hyperpigmented papules and plaques on the anterior region of the trunk (Figure 2A). A biopsy specimen revealed hyperkeratosis, papillomatosis, and a perivascular inflammatory infiltrate in the dermis with no signs of fungal infection, confirming the diagnosis of CRP. Within 1 month of doxycycline treatment (100 mg daily), lesions disappeared completely, with no relapse during 18 month-follow-up period (Figure 2B).

### Case 3

**Figure 3:**
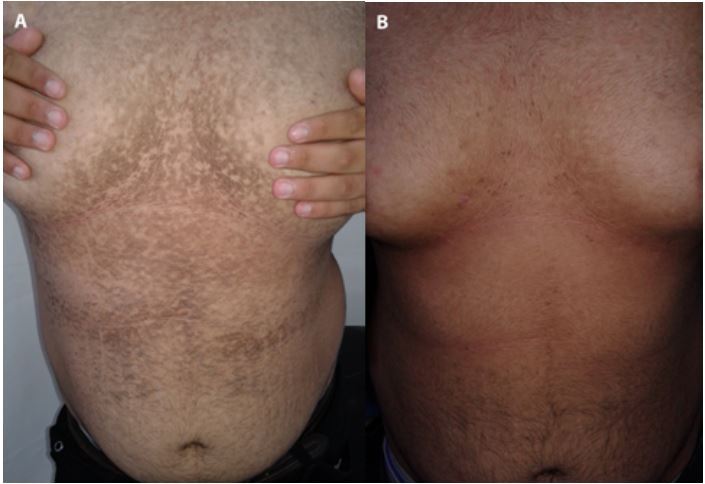
(A) Pigmented hyperkeratotic skin eruption on the trunk particularly on the intermammary region. (B) Complete clearance of lesions after 2 months of doxycycline.

A 16-year-old obese adolescent (BMI >35) presented with a 6-year history of asymptomatic, pigmented, hyperkeratotic papules and plaques around the neck and on the trunk (Figure 3A). Histological examination revealed orthohyperkeratosis, acanthosis, and papillomatosis associated with a normal dermis. A diagnosis of CRP was made and the patient was started on doxycycline (100 mg/day), with complete clearance of lesions in 2 months (Figure 3B). However, the patient was lost to follow-up.

## Conclusions

CRP is a rare dermatosis that affects young adults. Clinically, the eruption is characterized by the presence of pigmented hyperkeratotic papules and plaques generally asymptomatic and confluent in the center with reticular pattern at the periphery [1]. The lesions are typically localized to the trunk. The pathogenesis of CRP remains uncertain [1,2]. Some theories have suggested a disorder of keratinization, an involvement of bacterial pathogens via an alteration of sebum, or an abnormal response to host bacteria. Another hypothesis is endocrine disturbance based on an association of CRP with obesity, insulin resistance, diabetes mellitus, and other disorders of the thyroid and pituitary glands. To date, only 10 cases of CRP associated with obesity (including the presented cases) have been reported in the literature [2]. A pathogenic link between these 2 conditions is suggested by the insulin resistance and the resulting hyperinsulinemia in obese patients. High circulating insulin levels have mitogenic and anti-apoptotic activities on keratinocytes [2]. These activities may provide an explanation for the epidermal over-growth and papillomatosis seen in our patients. Concerning treatment, various modalities, mostly antibiotics, have been proposed with variable responses [1]. Complete resolution with doxycycline in our cases further support cyclins, safe and effective agents, as the treatment of choice for CRP. Their effectiveness is mainly attributed to their anti-inflammatory properties, most probably attributed to inhibiting neutrophil migration and subsequent reactive oxygen species release that inhibit matrix metalloproteinases, rather than antimicrobial effects alone.
